# Outpatient Management of Oligosymptomatic Patients with respiratory infection in the era of SARS-CoV-2: Experience from rural German general practitioners

**DOI:** 10.1186/s12879-020-05538-x

**Published:** 2020-11-06

**Authors:** Simon Wernhart, Tim-Henning Förster, Eberhard Weihe

**Affiliations:** 1 Sauerlandpraxis Medebach-Hallenberg-Winterberg, Niederstrasse 2, 59964 Medebach, Germany; 2 Institute of Anatomy and Cell Biology of the Philipps-University Marburg, 35037 Marburg, Germany

**Keywords:** General practitioner, COVID-19, Test strategy

## Abstract

**Background:**

Covid-19 is causing a pandemic and forces physicians to restructure their work. We want to share our experience in the outpatient management of potentially-infected patients with special consideration of altered national test strategies during the crisis.

**Methods:**

We analysed patients with respiratory symptoms reporting to our three rural general practitioner (GP) offices in North Rhine-Westphalia, Germany, from 27.01–20.04.2020 (*n* = 489 from a total of 6090 patients). A history of symptoms was taken at the doorstep following a specific questionnaire. Patients with respiratory symptoms were examined in a separated isolation area, while the others were allowed to enter the office. We applied the first recommended algorithm of the German Robert Koch Institute (RKI) to test suspected patients and compared our results with an adapted, more liberal version of the RKI, which is currently applied in Germany.

**Results:**

Eighty patients (16.36%, mean age: 47.03 years+ − 18.08) were sent to a nasopharyngeal smear. Five patients (6.25%) proved to be positive, four of whom had established risk factors for COVID-19. Overall, the most common symptoms were cough (83.75%), sore throat (71.25%), as well as myalgia and fatigue (66.25%). The most common diagnoses were rhinopharyngitis (37.22%) and acute bronchitis (30.27%). A *sore throat* was more common in positively-tested patients (80% vs. 12%). Applying the first RKI test strategy yielded 6.25% of positive tests (*n* = 80), while the more liberal later RKI recommendation would have achieved 1.36% positive tests from 369 patients. No positive test was missed by applying the conservative strategy. None of our employees called in sick during this period, which emphasises the efficacy and safety of our screening methods.

**Conclusion:**

A clinical distinction between ordinary respiratory infections and COVID-19 is not possible in a low-prevalence population. Our model to prevent unprotected physical contact, screen patients in front of the office with protective equipment, and examine respiratory infections in separated areas works in the GP setting without overt health risks for employees. Thus, this approach should be used as a GP standard to uphold patient care without major health risks for the personnel. Large multi-centre studies are necessary to work out the most suitable test strategy.

## Background

Originating from Wuhan, China, Corona Virus Disease 2019 (COVID-19) has spread around the world as a pandemic and created enormous health, political, and economic problems [1, 2], with more than 18 million confirmed cases worldwide and more than 700,000 deaths as of 07.08.2020 [3].

Clinical predictors of mortality have been suggested from a cohort of patients from Wuhan [4–6] and recommendations for outpatient [7] and inpatient care [6] as well as intensive care treatment [8] have been proposed. Patients admitted to intensive care units are older, and tend to have higher leukocyte counts, D-Dimers, LDH, creatinine, and troponin levels [5]. Elevated troponin as a marker for myocardial injury heralds a poor prognosis [9].

The most common symptoms – depending on the time window during the course of infection – are symptoms associated with respiratory diseases, such as fever, cough, sore throat, headache, chills, fatigue and myalgia, smell and taste dysfunction and gastrointestinal problems [10, 11], but recently more severe cases have been associated with neurological symptoms, such as acute cerebral vascular disease, skeletal muscle injury and impaired consciousness [12]. Perniosis-like skin symptoms may also be present [13, 14]. Risk factors for more severe cases of COVID-19 are hypertension, coronary artery disease, immunosuppression, and chronic lung disease [15].

Less severe cases often present to the general practitioner (GP) in the outpatient setting, which requires precautions to avoid infection among medical staff. In Germany, testing for SARS-CoV-2 infection has been widely established [16, 17]. Additionally, containment as a means to reduce exponential growth has been implemented at an early stage [18], which may account for the relatively low German case mortality rate in March and April 2020 (4.1%) compared with other European countries [19, 20]. As of 07.08.2020, in Germany more than 200,000 patients had been infected and more than 9000 had died [3]. Furthermore, standard and elective examinations have been postponed to limit physical physician-patient contact to the necessary minimum [21].

The German government as well as national and federal medical institutions have made considerable efforts to prevent less critical but potentially infectious patients from showing up in the GP office by installing telephone and video conference facilities to provide medical council without physical contact. However, in practice concerned patients who fear that they are infected keep showing up and need to be screened in isolation rooms in case of symptoms suggestive of COVID-19. This requires office re-organisation and efforts to obtain personal protective equipment (PPE), albeit which has been difficult to access for some time.

The German Robert Koch Institute (RKI) has issued recommendations for COVID-19 screening in the outpatient setting [22]. However, a differentiation between oligosymptomatic COVID-positives and ordinary infections seems almost impossible. Here, we present real-life data from our three large rural GP offices in North Rhine-Westphalia, Germany, between 27.01 and 20.042020 to demonstrate the difficulty of filtering oligosymptomatic patients with low pre-test probability. Our aim is to share our experience as GPs with colleagues from other countries, where infection rates may be even higher and viral doubling time is very low.

## Methods

**Fig. 1:**
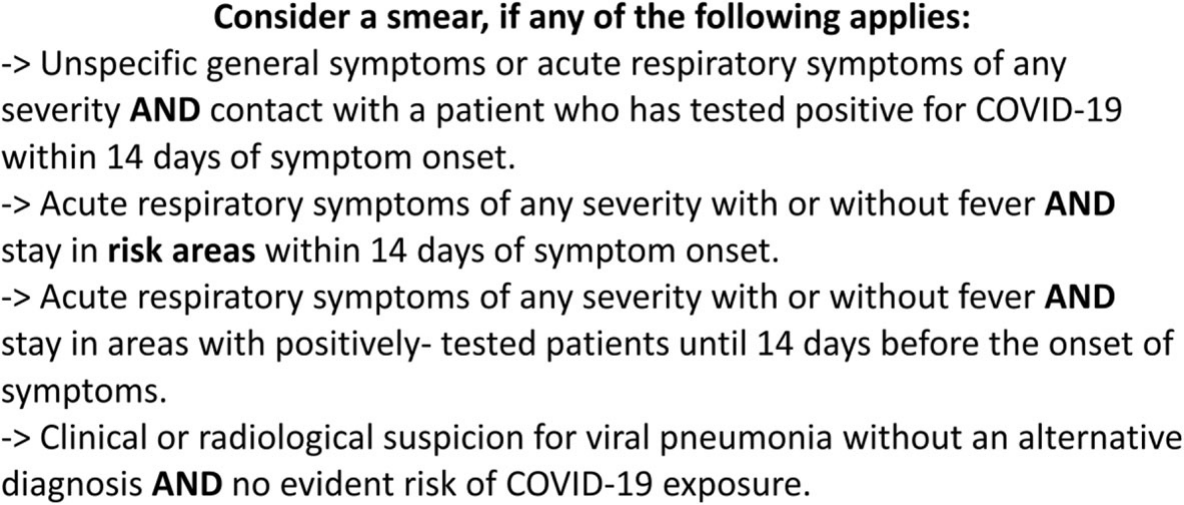
First test criteria of the RKI. SARS-CoV-2 test criteria of the German Robert Koch Institute as of March 2020

We analysed data from our three GP offices in rural North Rhine-Westphalia, Germany, from 27.01.2020 until 20.04.2020 and selected all patients reporting symptoms of respiratory tract infection. According to the RKI guidelines during the study period to screen patients for potential COVID-19 infection (Fig. 1), we chose to either send patients to a nasopharyngeal smear or treat them conservatively. Due to the limited availability of smear testing in our rural area at that time, we were unable to perform the smears in our offices, but had to transfer patients to the local hospital. Patients were put into quarantine until the results of the tests were available. We have gained satisfactory experience with the following procedures and algorithm at the onset of the pandemic in Germany: one GP with PPE comprising a gown, goggles, caps, gloves and an FFP (filtering face piece)-3 mask screens every patient in front of our office, asking the following questions:

1. Are you currently suffering from a cough or sore throat?
2. Have you measured a temperature > 38.5 degrees Celsius in the last five days?
3. Have you had direct contact with a person who has tested positive for COVID-19?
4. Are you employed in a medical profession?
5. Are you suffering from a loss of smell or taste?
6. Are you suffering from myalgia, fatigue or headache?
7. Are you suffering from diarrhoea or vomiting?
8. Are you suffering from immunosuppressive disease?

If two of these questions were answered with *yes*, patients were directed to an isolation room, which was supplied with all basic medical devices to provide a fast clinical exam. Patient history was documented vigorously. In case of persisting suspicion following the diagnostic algorithm of the RKI for COVID-19 [22], patients were directly referred to our smear centre. If an ordinary respiratory infection was given as a diagnosis, people were instructed on general hygiene recommendations and treated conservatively. Only patients with symptoms not suggestive of respiratory disease were allowed to enter the regular office.

**Fig. 2:**
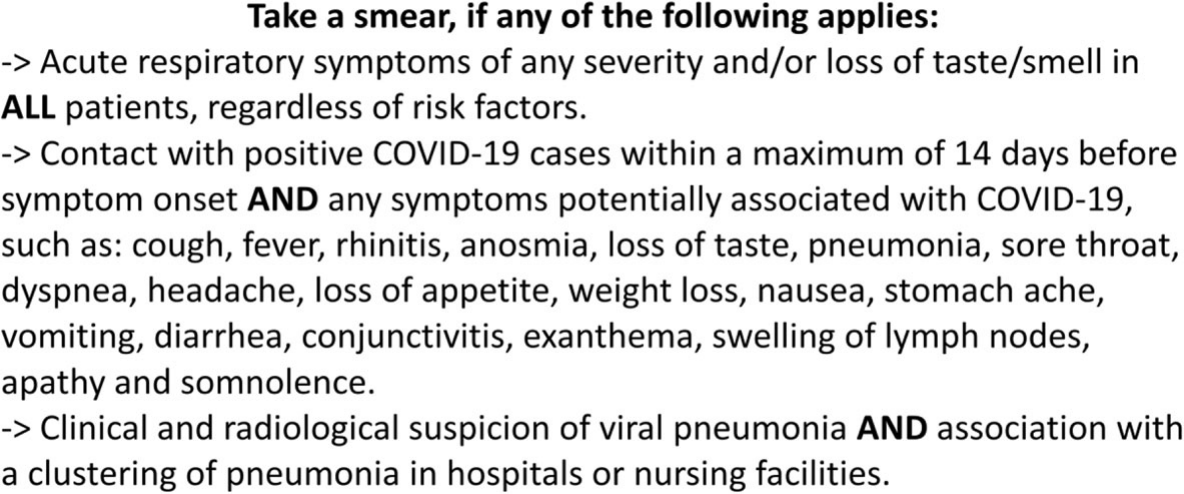
Modified test criteria of the RKI. SARS-CoV-2 test criteria of the German Robert Koch Institute as of August 2020

There was a change in RKI guidelines on how to screen outpatient contacts after the end of our study (Fig. 2). We therefore calculated the number of recommended smears for the first and modified RKI guidelines.

## Results

**Fig. 3:**
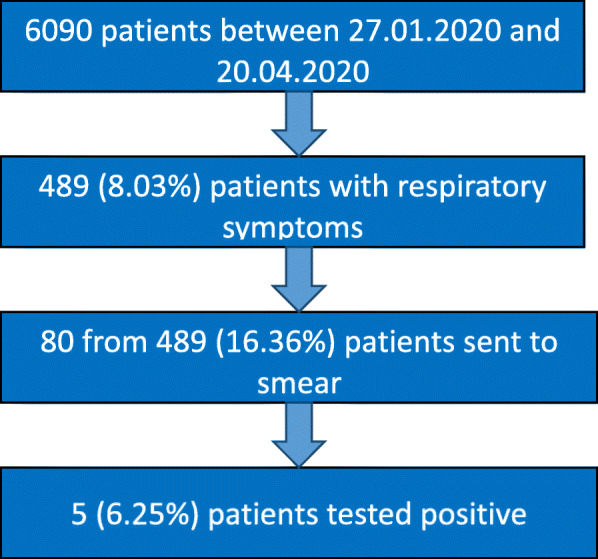
Data collection. Description of the process of data collection (percentages shown in parentheses)

The mean age of all tested patients (*n* = 80) was 47.03 years + − 18.08 (mean age of positively tested, 50.20 years+ − 13.76; *n* = 5), while the mean age of all symptomatic patients (*n* = 489) was 52.69 years+ − 14.75. Symptoms across all respiratory infections are provided in Table 1, while Fig. 3 illustrates the data collection. 13.75% of patients with respiratory tract infections were recent returners from (at that time) risk areas of transmission defined by the RKI (mainly from Austria and the Netherlands). 8.75% had had significant (at least 15 min) contact with a person who had tested positive for COVID-19.

**Table 1:** Symptoms of patients with respiratory tract infections

Symptoms in all patients (***n*** = 489)	Symptoms: negatively tested (***n*** = 75)	Symptoms: positively tested (***n*** = 5)
Cough *n* = 407 (83.23%)	Cough *n* = 63 (84.00%)	Cough n = 4 (80.00%)
Sore throat *n* = 70 (14.35%)	Sore throat *n* = 9 (12.00%)	Sore throat n = 4 (80.00%)
Myalgia and fatigue *n* = 309 (63.19%)	Myalgia and fatigue *n* = 50 (66.67%)	Myalgia and fatigue n = 3 (60.00%)
Headache *n* = 158 (32.31%)	Headache *n* = 22 (29.33%)	Headache n = 2 (40.00%)
Rhinitis *n* = 245 (50.10%)	Rhinitis *n* = 20 (26.67%)	Rhinitis n = 3 (60.00%)
Fever > 38.5 degree Celsius n = 70 (14.31%)	Fever > 38.5 degree Celsius n = 9 (12.00%)	Fever > 38.5 degree Celsius *n* = 1 (20.00%)
Smell and taste dysfunction *n* = 51 (10.43%)	Smell and taste dysfunction n = 9 (12.00%)	Smell and taste dysfunction *n* = 0 (0.00%)
Chills *n* = 69 (14.11%)	Chills n = 8 (10.67%)	Chills n = 1 (20.00%)
Earache *n* = 41 (8.38%)	Earache n = 4 (5.33%)	Earache n = 0 (0.00%)

Table 1. Symptoms associated with respiratory tract infection between 27.01.2020 and 20.04.2020 in all patients (*n* = 489), those negatively tested (*n* = 75) and positively tested (*n* = 5).

By far the most common diagnoses among the entire patient population were rhinopharyngitis and acute bronchitis, while pneumonia was less commonly found (Table 2). Due to the low number of positive tests, we did not perform mean comparison tests, and data were depicted as absolute values and percentages.

**Table 2:** Diagnoses of patients reporting with respiratory tract infections

Diagnosis in all patients (***n*** = 489)	Diagnosis: negatively tested (***n*** = 75)	Diagnosis: positively tested (***n*** = 5)
Rhinopharyngitis: *n* = 182 (37.22%)	Rhinopharyngitis *n* = 23 (30.67%)	Rhinopharyngitis n = 2 (40%)
Acute bronchitis *n* = 148 (30.27%)	Acute bronchitis *n* = 25 (33.33%)	Acute bronchitis *n* = 3 (60%)
Acute sinusitis *n* = 52 (10.64%)	Acute sinusitis *n* = 19 (25.33%)	Acute sinusitis *n* = 0 (0%)
Tonsillitis *n* = 34 (6.95%)	Tonsillitis *n* = 3 (4.00%)	Tonsillitis *n* = 0 (0%)
Otitis media *n* = 34 (6.95%)	Otitis media *n* = 2 (2.67%)	Otitis media *n* = 0 (0%)
Pneumonia *n* = 31 (6.33%)	Pneumonia *n* = 3 (4.00%)	Pneumonia *n* = 0 (0%)
Laryngitis *n* = 8 (1.64%)	Laryngitis *n* = 0 (0%)	Laryngitis *n* = 0 (0%)

Table 2. Distribution of diagnoses in all patients reporting with signs of respiratory infection (*n* = 489), those negatively tested (*n* = 75) and positively tested (*n* = 5) between 27.01.2020 and 20.04.2020. A diagnosis of pneumonia was established in case of significant auscultation and one additional symptom, such as fever or productive sputum. Chest X-ray was not available.

Among 80 patients who met the first (Fig. 1) RKI criteria [22] and were sent to the smear centre, only five proved to be positive. The most common symptoms in positive patients were cough (4/5), sore throat (4/5), myalgia and fatigue (3/5) and rhinitis (3/5). Headache (2/5), chills (1/5) and fever (1/5) were less common. None of these positively-tested patients suffered from smell or taste dysfunction or ear ache. According to the modified RKI criteria (Fig. 2), we would have sent 369 patients to the smear centre (75.46%), also gaining five positive test results (1.36%). During the study period, we did not experience any patients who were not sent for a smear first but were tested positive later on.

Table 3 illustrates the comorbidities of positively- and negatively-tested patients. In both groups, no skin alterations were detected.

**Table 3:** Prevalence of comorbidities in patients tested for SARS-CoV-2

Tested positive	Tested negative
Diabetes: *n* = 2 (40.00%)	Diabetes: n = 2 (2.67%)
Arterial hypertension: n = 3 (60.00%)	Arterial hypertension: n = 23 (30.67%)
Hypothyroidism: n = 0 (0%)	Hypothyroidism: *n* = 11 (14.67%)
Immunosuppression: n = 1 (20.00%)	Immunosuppression: *n* = 6 (8.00%)
Atrial fibrillation: *n* = 1 (20.00%)	Atrial fibrillation: *n* = 5 (6.67%)
Coronary artery disease: n = 0 (0%)	Coronary artery disease: n = 3 (4.00%)
Lung disease: n = 2 (40.00%)	Lung disease: *n* = 10 (13.33%)
Depression: n = 1 (20.00%)	Depression: n = 11 (14.67%)
Chronic kidney disease: n = 0 (0%)	Chronic kidney disease: n = 2 (2.67%)
RAAS inhibitors: n = 2 (40.00%)	RAAS inhibitors: n = 15 (20.00%)
Oral anticoagulation: n = 1 (20.00%)	Oral anticoagulation: n = 5 (6.67%)
Platelet inhibitors: n = 1 (20.00%)	Platelet inhibitors: n = 1 (1.33%)

Table 3. Prevalence of comorbidities in patients tested positive (*n* = 5) and negative (*n* = 75) for COVID-19. Immunosuppression was defined as autoimmune disease or cancer in patient history. Lung disease was defined as chronic obstructive lung disease or asthma under medical treatment. Chronic kidney disease was defined as a glomerular filtration rate (GFR) < 60 ml/min for at least three months. Oral anticoagulants included vitamin K-antagonists (VKA) and new oral anticoagulants (NOAK.), platelet inhibitors included Aspirin, Clopidogrel, Ticagrelor or Prasugrel. RAAS inhibitors: renin-angiotensin-aldosterone system inhibitors.

## Discussion

We analysed data from our three GP offices in rural Germany between the onset of Covid-19 in our country on 27.01.2020 until 20.04.2020. The mean age of our patients was 47.03 years, which is quite young considering that mortality seems to increase in COVID-19 patients beyond 65 years, while patients younger 65 with minimal predisposing factors may be at a low risk of severe disease [5].

Only five out of 80 tested patients were positive for SARS-CoV-2 (positives). Due to this low number and a potential reporting bias of symptoms, we refrained from using mean comparison tests and only depicted absolute values. However, we noticed that almost all patients in the positively-tested group suffered from a sore throat (4/5; 80%), while only 12% in the negatively-tested group (negatives) showed this symptom (9/75). Furthermore, rhinitis was more prominent in the positively-tested group (60% vs. 26.67%). Due to the limited case number, generalisability of these findings is not possible.

Known comorbidities – especially pre-existing lung and cardiovascular disease – in positively- and negatively-tested group were quite low (see Table 3). The most common cardiovascular risk factor was arterial hypertension, which has already been published [5]. SARS-CoV-2 uses ACE-2 as a cellular entry point [23] and has raised concerns about the continuation of RAAS inhibitor intake in patients with chronic heart failure [24]. However, recent data has shown that there is no evidence of increased disease severity or mortality in hospitalised patients on RAAS blockers [25, 26]. Additionally, pharmacological data suggests that ACE-2 expression is not increased in patients on RAAS blockers [27]. Thus, current recommendations support the continuation of RAAS blockers in patients with arterial hypertension and chronic heart failure [24]. In our study, two out of five positively-tested patients were on RAAS blockers and did not display more severe symptoms than the others. Moreover, among the negatively-tested group RAAS blockers were the most commonly-prescribed antihypertensive drugs (15 out of 23 patients received RAAS blockers), showing no difference in clinical severity.

We applied the first algorithm (Fig. 1) provided by the RKI [22] to decide which patients needed a smear. 6.25% of the tested patients were positive. This algorithm was introduced at the beginning of the crisis, during a time when travel restrictions had not been implemented across Europe. There was limited knowledge on the diversity of symptoms associated with SARS-CoV-2 at the time, and therefore unspecific respiratory symptoms alone did not qualify to send a patient to a smear centre. Travelling to a risk area and contact with positively-tested patients were important criteria in the first RKI definition.

Schmithausen et al. found that early temporary loss of taste and smell occurred in 68% of oligosymptomatic patients who tested positive for SARS-CoV-2 after a cluster outbreak at a carnival celebration in North Rhine-Westphalia, Germany, less than 100 km away from our offices [28]. Similar to our study (80%), the most common symptom was a dry, non-productive cough (73%). Consequently, the RKI adapted their recommendation for SARS-CoV-2 diagnostics (after the end of our study) and implemented *loss of taste and smell* as a paramount factor into their algorithm (Fig. 2). It should be noted that acute respiratory symptoms of any severity now qualify to take a smear, and thus the number of smears will increase. In our population, this adapted RKI algorithm would have detected all five positive patients, but it would have also produced a much larger number of tested individuals (*n* = 369) and significantly higher costs for local health authorities. 1.36% of tested patients would have been positive, as opposed to 6.25% following the first RKI recommendations. Additionally, due to a reliable recall system from our offices, the clinic with the smear centre, and the health department, we were able to confirm that none of the patients who tested negative for SARS-CoV-2 subsequently tested positive at a later stage during the study period. This might suggest that the more conservative first RKI algorithm would have been sufficient in our low-prevalence population and the adapted, more liberal algorithm may have led to over-testing and higher costs. Our five patients who tested positive were kept in quarantine for two weeks, by when their symptoms had subsided. Therefore, further transmission after release is highly unlikely.

During our study, initial national efforts were devoted to measures to make testing available to everyone, especially in rural areas such as ours. During the study period, we had to send all patients to the local smear centre, which changed in May 2020 when we started performing smears directly in our offices.

A more focused approach of smear-taking has been used since the advent of clustered outbreaks [28], with a large number of positive tests along with a very low number of positive tests, such as in our area. The debate on the *ideal smear strategy* is ongoing. Our data contributes essential input to this debate because we can show that intensifying the smear strategy – as was done by the German RKI – does not yield a better detection of positive cases in low-prevalence populations. Perhaps algorithms have to be adapted to the federal or even local prevalence as well as regional cluster outbreaks. A very liberal strategy also binds human resources in the GP offices, which can create new problems.

Another issue that has not been entirely solved is sequential testing: a median incubation period of five days was estimated [29], which involves the danger that a negative test during early infection may pretend a false safety. Sequential testing may circumvent this, but was not applicable in our study.

In Germany, we have a health system based on solidarity, in which most people have health insurance and thus have easy access to health care. As the first medical contacts, GPs have to filter many patients directly in the office. The Center for Disease Control (CDC) has issued similar recommendations for the public as the RKI in Germany, namely (1) to cover one’s own mouth and nose with a cloth, (2) call the GP’s office first instead of showing up directly, (3) avoid entering into close physical contact with others, (4) engage in regular disinfection of one’s hands and surfaces, (5) and self-monitor symptoms [30]. The paramount aim of medical personnel in this crisis is to maintain optimal medical care and personal health in a high-risk environment. Thus, stringent algorithms for GPs and other medical specialties need to be introduced to achieve this goal. By segregating patients who are suggestive of respiratory infection and treating them under high standards of hygiene and protection, we believe that we have achieved this goal in a practicable and efficient manner. None of our employees called in sick during the study period, which may suggest that our stringent selection process prior to entering the office is a success. Our GP experience could now help colleagues in other countries with a later onset of COVID-19 than Germany to organise their offices with the available resources. The transparency of strategies from different countries on how to deal with COVID-19 in inpatient and ambulatory settings holds paramount importance to optimise further patient care and improve educational measures [31].

Our data shows that it is not possible to accurately differentiate between oligosymptomatic COVID-19 patients and ordinary respiratory infection by analysing symptoms alone. Recently, Arons et al. published about the spread of COVID- 19 in a US nursing home, in which more than half of positively-tested patients were asymptomatic [32, 33]. This clearly demonstrates that strategies focusing only on symptoms fail to prevent further transmission.

There is a conceivable limitation to our study: although we have investigated a large number of patients, we live in a low-prevalence area, which excludes inductive statistics and allows only a description of data. The validity of both suggested algorithms from the RKI has to be analysed in a larger and more diverse cohort. Large, prospective, randomised multi-centre trials will be necessary to ascertain the most suitable test strategy.

To sum up, our study illustrates that the application of the modified and more liberal RKI algorithm (Fig. 2) to filter patients with minor respiratory tract infections in the GP office in a low-prevalence area would lead to more (negative) testing than the first, more conservative version (Fig. 1). Different results may be gained in high-risk areas. We did not observe a conceivable difference in symptoms between COVID-19 patients and patients with ordinary respiratory tract infection. Our study also adds valuable information to the literature on how to practically manage patient care in a GP office during the peak of the COVID crisis in Germany in 2020. Additionally, we would suggest integrating working in a high-risk profession (such as hospitals or nursing homes) as a factor for future risk assessment scores for COVID-19 infections. Furthermore, as an easily-available tool in outpatient care, lung ultrasound could help to better detect COVID-19, since it has been shown that B-lines – a typical sonographic sign of pneumonia – are present at an early stage of the disease [34].

## Conclusion

In summary, we provide real-life data from rural GP offices in Germany demonstrating the difficulty of distinguishing oligosymptomatic COVID-19 patients from ordinary respiratory tract infection. We provide a well-working example on how to re-organise a GP’s office to separate potentially infectious patients from the rest with minimal risk of further spreading the disease.

## Data Availability

The datasets analysed during the current study are not publicly available, but will be made available from the corresponding author on reasonable request.

## Abbreviations

ACE-2: Angiotensin converting enzyme
CDC: Center for Disease Control
COVID-19: Corona Virus Disease 2019
FFP-3: Filtering face piece
GFR: Glomerular filtration rate
GP: General practitioner
PPE: Personal protective equipment
RAAS: Renin-angiotensine-aldosterone system
RKI: Robert Koch Institute
SARS-CoV-2: Severe acute respiratory syndrome- Corona virus 2
VKA: Vitamin K antagonist
